# Optimization of therapeutic antibodies

**DOI:** 10.1093/abt/tbab003

**Published:** 2021-02-18

**Authors:** Bo Wang, Sachith Gallolu Kankanamalage, Jianbo Dong, Yue Liu

**Affiliations:** Ab Studio, Inc. Hayward, CA 94545, USA; Ab Studio, Inc. Hayward, CA 94545, USA; Ab Studio, Inc. Hayward, CA 94545, USA; Ab Studio, Inc. Hayward, CA 94545, USA; Ab Therapeutics, Inc. Hayward, CA 94545, USA

**Keywords:** antibody therapy, antibody optimization, humanization, affinity, maturation, computer-aided design, antibody safety, antibody efficacy, antibody developability

## Abstract

In this review, we have summarized the current landscape of therapeutic antibody optimization for successful development. By engineering antibodies with display technology, computer-aided design and site mutagenesis, various properties of the therapeutic antibody candidates can be improved with the purpose of enhancing their safety, efficacy and developability. These properties include antigen binding affinity and specificity, biological efficacy, pharmacokinetics and pharmacodynamics, immunogenicity and physicochemical developability features. A best-in-class strategy may require the optimization of all these properties to generate a good therapeutic antibody.

**Statement of Significance:** Development of therapeutic antibodies usually go through multiple steps of optimization process, in order to reach the best balance on safety, efficacy, manufacturability and have the subsequent clinical success. In addition to traditional antibody engineering technologies, newly emerging computer-aided approaches are evolving the ways the therapeutic antibody lead optimization processes are performed.

## INTRODUCTION

Therapeutic antibodies have become an important option in treating numerous diseases. Since the first therapeutic antibody Orthoclone Okt3 was approved by the Food Drug Administration in 1986, ~100 monoclonal antibodies (mAbs) and three bispecific antibodies have been designated as drugs [1]. The field of therapeutic antibodies is becoming increasingly competitive, whereas the development cost of a successful therapeutic antibody is also increasing with time. Therefore, identifying novel methods to optimize the safety, efficacy and manufacturability of antibody candidates would be critical to the efficient development of therapeutic antibodies.

**Figure 1:**
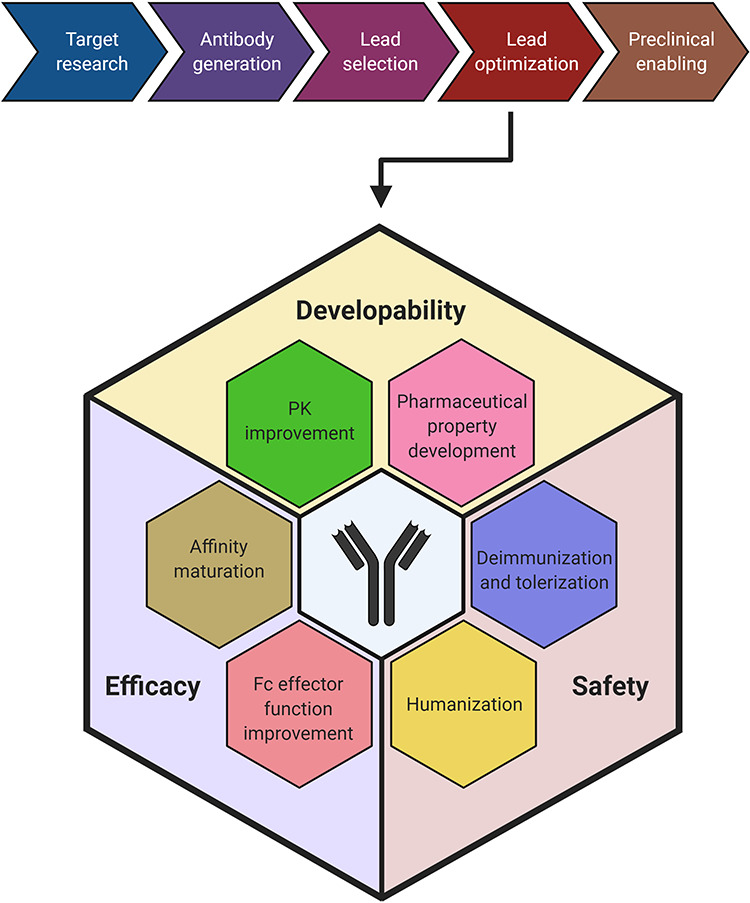
Preclinical development workflow of therapeutic antibodies with an emphasis on lead optimization. The therapeutic antibodies undergo five major sequential steps during the preclinical development, namely target research, antibody generation, lead selection, lead optimization and preclinical enabling. This review has focused on the lead optimization step. Therapeutic antibody optimization is performed to improve their safety, efficacy and developability features. The strategies of humanization and deimmunization and tolerization are performed to enhance the safety, whereas affinity maturation and Fc effector function improvement are performed to enhance efficacy. The pharmacokinetic improvement and pharmaceutical property development are performed to improve the developability features of the antibodies.

**Figure 2:**
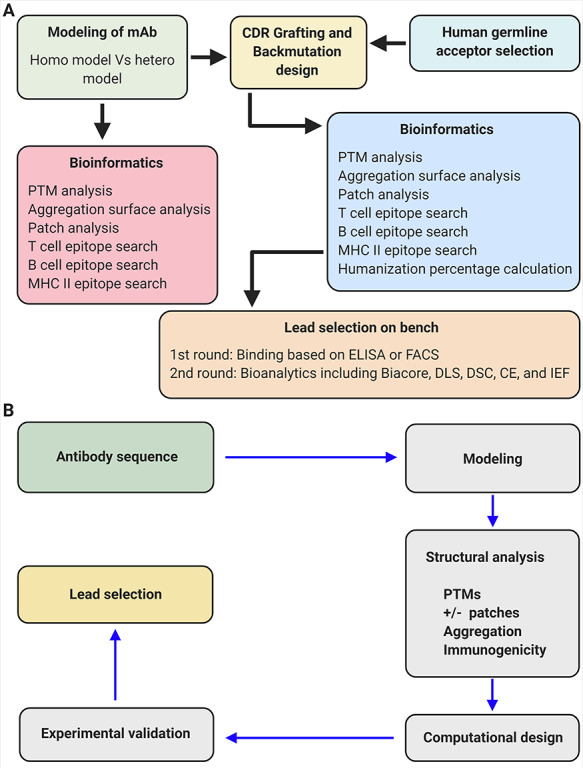
The workflow of antibody humanization and optimization and how the computer-aided antibody design contributes to this process. (A) The workflow of antibody humanization and optimization. (B) The workflow of computer-aided antibody design.

Therapeutic antibody candidates usually have to undergo the following research and development phases before entering clinical studies: antibody discovery and screening based on the antigen binding ability, lead selection based on biological function and lead optimization to enhance the balance among safety, efficacy and manufacturability. In this review, we will mainly focus our discussion on the step of lead optimization (Fig. 1). It should be noted that Figure 1 depicts the relationships between antibody optimization strategies and optimization purposes in a simplified manner. However, some of the optimization strategies could serve multiple purposes. For example, deimmunization could improve both the safety and efficacy features of a therapeutic antibody. The simplified workflow of lead optimization and how the computer-aided antibody design assists in this process are shown in Figure 2.

## OPTIMIZATION OF THERAPEUTIC ANTIBODIES

Reduction of a nonhuman antibody’s immunogenicity in humans is a critical step of antibody optimization; otherwise the therapeutic antibody candidate would induce anti-drug antibodies in humans. For this purpose, antibody humanization and deimmunization strategies need to be performed.

## ANTIBODY HUMANIZATION

In order to reduce the immunogenicity, variable region of a nonhuman antibody can be fused to human antibody constant region to generate a chimeric antibody. To further reduce the immunogenicity, the variable region of the chimeric antibody could be modified to increase its similarity to antibody variants produced naturally in humans to generate a ‘humanized antibody’. This modification process is called ‘humanization’. Antibody humanization is usually performed by studying the difference between the nonhuman antibody sequence and that of its human homologs. On each position where the nonhuman residue and human residue are different, a selection needs to be made. If application of the human residue will not affect the binding affinity of the antibody or significantly reduce the ‘developability’ of the antibody, human residue is selected. Otherwise, mouse residue is maintained (maintenance of mouse residue is also called ‘back mutation’).

Multiple methods are used for the antibody humanization process. Computer-aided design, phage display and yeast display are three widely used approaches for humanization. While computer-aided design allows the user to construct a 3D homology model structure, analyze the structure and design mutations *in silico*, phage display and yeast display allow them to physically assess all possibilities to determine whether a mouse or human amino acid residue should be selected at each testing position. If only the framework region is modified in the antibody, that humanization process is called complementary determining region (CDR) grafting, whereas if both framework and CDR regions are modified while the specificity determining residues (SDR) that directly interact with the antigens are unchanged, it is called SDR grafting [2–4].

While well-known software used for computer-aided design such as BioLuminate and MOE continuously update their platforms to make the applications more user friendly, new tools are also emerging in the field. For instance, a new webserver, Tabhu, is developed with tools for human template selection, grafting, back mutation evaluation, antibody modeling and structural analysis. In contrast to many other computer-based tools, Tabhu screens and selects the human framework donor sequence with highest similarity to the xenogeneic V region from a database with two sources: 1) Digit database including the sequences of both light chain and heavy chain; 2) IMGT's human germline gene sequences. The selected Variable and Joining genes, and the mouse CDRs, can be assembled together to form the initial antibody molecule. Tabhu also has a new function, proABC, used for CDR grafting and affinity prediction, in addition to procheck and EDTSurf that alert the user when the introduction of back-mutations generates cavities or clashes [5].

## ANTIBODY DEIMMUNIZATION AND TOLERIZATION

Though humanization of a therapeutic antibody candidate is one approach to render biologicals less foreign to the human immune system, fully humanized mAbs may still display immunogenicity. While numerous factors such as aggregation, dose, route and target can contribute to the immunogenicity of therapeutic antibodies, one of the key contributors to this effect is the epitope sequences contained within the antibody. The major antigenicity epitopes include the T cell, B cell and MHC epitopes and other antigenicity epitopes. Deimmunization is the process of identifying and removing these epitopes. Multiple computational tools, for example Protean 3D from DNA star, are used to predict these epitopes in the query sequences. Because most of these tools cannot determine whether the predicted epitope is displayed on the protein surface (which will be detected by human immune system) or not, the prediction for a ‘3D’ epitope alone, such as a B cell epitope, usually is insufficient. Therefore, combination of information from linear sequence prediction and percentage of displaying of the epitope on a 3D model is a better way to identify immunogenicity epitopes.

Other than removing immunogenicity epitopes of a therapeutic antibody candidate, another way to repress its human immunogenicity is to induce immune-tolerization. This new approach is performed by introducing Treg epitopes into the antibody structure. The Treg epitopes would stimulate Treg cell functions and provide the antibody with immune tolerance. This approach serves as a means of improving biologics’ ‘quality by design’ and may lead to the development of highly clinically effective but less immunogenic therapeutic antibodies [6].

The next step in the therapeutic antibody optimization process is to improve the antibody’s efficacy. Affinity maturation is a routine method used for this purpose to improve the binding of an antibody to its target antigen.

## ANTIBODY AFFINITY MATURATION

Antigen binding affinity is one of the most critical properties of a therapeutic antibody. Therefore, methods used for antibody affinity maturation, including random mutagenesis, targeted mutagenesis, chain shuffling and *in silico* approaches, are widely applied. The first three methods are usually performed by using display technologies, such as phage display. *In silico* approach is based on computer-aided design, and this is a newer approach compared with the others. BioLuminate is one of the software packages which contain ‘affinity maturation’ function. In order to optimize the binding affinity of an antibody, a 3D structure of an antibody–antigen complex needs to be analyzed. If there is no crystal structure available for the antibody–antigen complex in computational databases, generating a homology model of the complex should be performed prior to the analysis. This can be achieved either by docking a peptide antigen into a 3D homology antibody model via ‘molecule docking’ or by docking the antigen homology model into an antibody homology model via ‘protein–protein docking’. While the affinity maturation calculations could be performed with the docking models, the accuracy of this approach may vary from case to case.

Antibody-antigen interaction usually involves multiple non-covalent interactions. Although the calculation of protein-protein binding energy remains a challenging task, computational design of biotherapeutic molecules has made big progress thanks to the new computing capacities and algorithm. In a 2014 report [7]. Kiyoshi *et al*. employed structure-based computational design to inprove the affinity of 11K2 antibody. The single-mutation variant with highest affinity has 4.6 fold higher affinity than the parental clone, which already has a very high affinity of 4.6 pM. Interestingly, all the single mutation showing increased affinity has mutated to a charged residue. In another study. For instance, Lippow *et al*. [8] reported that they had achieved over 100-fold improvement of antigen binding affinity in an antibody by performing *in silico* affinity maturation. After combining multiple designed mutations into one engineered antibody, the anti-EGFR drug cetuximab (Erbitux) obtained a 10-fold affinity improvement (to 52 pM) , and the anti-lysozyme model antibody D44.1 obtained a 140-fold improvement in affinity (to 30 pM). The results of this work show that computed electrostatics alone works better than the computed total free energy in predicting the binding improvement. Electrostatic-based predictions yielded fewer false positives and more true positives. Additional groups have exploited the designability of electrostatic interactions in the antibody–antigen interaction interface, further demonstrating that it is more predictable and effective in *in silico* affinity maturation process [7,9].

So far, most of the *in silico* affinity maturation works have relied on the availability of crystal structures of the antibody–antigen complexes. But very often these structures are not available. Recently, Cannon *et al*. [10] reported an example of successful *in silico* affinity maturation of mouse antibody, AB1, by using only a homology model of the antibody variable region and a antibody-antigen docking model of the AB1 antibody and it antigen (muCCL20). A 3D model of AB1 and a crystal structure of muCCL20 were used to generate the protein–protein docking model. To narrow down the predicted docking poses, the authors exploited the fact that AB1 does not bind to human CCL20 and is able to block the interaction of muCCL20 with its cell surface–expressed receptor CCR6. The authors further refined and re-docked the interactions based on the *in silico* and experimental alanine scanning results. Three separate *in silico* algorithms were employed to perform the mutagenesis, which yielded a panel of 20 variants that were subjected to validation by subsequent assays. Two of the tested variants were shown to have a 3–4-fold affinity improvement.

While the progress shows encouraging signs, *in silico* affinity maturation process still faces a lot of challenges, including interfacial-trapped water molecules, conformational changes upon binding, and the trade-off of protein–solvent with protein–protein interactions from the unbound to bound state. In addition, redesigning from nanomolar to picomolar affinities remains a particular challenge [8].

In order to reduce the cross reactivity of an antibody to other antigens or to broaden the specificity of an antibody to related antigens or to improve an antibody’s cross-species binding ability, the antibody needs to be engineered to optimize its specificity.

## ANTIBODY SPECIFICITY OPTIMIZATION

Random mutagenesis and targeted mutagenesis are two common approaches for antibody specificity optimization. A potential new approach for this purpose is *in silico* design of those antibodies with mapped epitopes. For instance, if the 3D structure of an antibody–antigen complex is available, the mutation of the human epitope to mouse epitope (assuming human and mouse antigens share a certain homology) would generate a new 3D model structure of antibody–mouse antigen. This process can be performed by using ‘affinity maturation function’ in computational platforms such as BioLuminate, and the predicted antibody mutations with improved mouse antigen binding can be tested on bench. Any mutants that do not weaken the binding to the human antigen but have improved binding to the mouse antigen would be successful human–mouse cross-species binders.

A previous case study has implied that electrostatic interactions at the binding site may play an important role in specificity and cross-reactivity [11]. The highly overlapping epitopes on hen egg-white lysozyme (HEL) are recognized by the antibodies HyHEL8, HyHEL10, and HyHEL26 with similar affinities, despite having different specificities. The binding sensitivity of these antibodies towards the epitope mutations increases in the order of HyHEL8, HyHEL10, and HyHEL26, respectively. Therefore, HyHEL8 is the most cross-reactive, whereas HyHEL26 is the most sensitive of these antibodies. Using these antibodies as model molecules, the authors demonstrated that higher electrostatics (as a number of short-range electrostatic interactions and their contributions) cause greater specificity in binding. Strong salt bridges, salt bridge networking, and electrostatically driven interactions cause geometric constrains that ultimately result in the limitations of binding flexibilities. However, the hydrophobic interactions and lower amounts of electrostatic interactions result in conformational flexibility in molecules and their subsequent cross-reactivity. This knowledge could be used in engineering the binding specificities of the therapeutic antibodies.

Other than improving the antigen binding ability of an antibody, which is mainly determined by the variable region of IgG, the biological efficacy of an antibody can also be modulated by Fc engineering.

## ANTIBODY EFFICACY IMPROVEMENT

It has been well established that IgG antibodies coating on pathogens can evoke the immune effector functions such as antibody dependent cell-mediated cytotoxicity (ADCC), antibody induced complement dependent cytotoxicity (CDC), and antibody dependent cell-mediated phagocytosis (ADCP) [12,13]. While CDC is mediated and initiated by the binding of Fc domain to the first component of complement, C1q [14], ADCC and ADCP are executed by the innate immune cells such as natural killer cells and macrophages. The Fc gamma receptors (FcγR) on their surfaces are capable in recognizing and binding to the Fc domains, and as a result regulate antibody-dependent effector functions by the cells. Human cells possess six different FcγR: hFcγRI, hFcγRIIA, hFcγRIIB, hFcγRIIC, hFcγRIIIA and hFcγRIIIB [15].

As early as in 1988, Greg Winter group has identified G235 in a mouse IgG2b antibody as a key binding site for FcγRI by oligonucleotide site-directed mutagenesis [12]. E235L mutation results in a 100-fold improvement in affinity. From then on, a large body of work has focused on studying the effects of Fc variants. In 2001, Shields *et al*. performed high resolution mapping of the FcγR binding sites on human IgG1 Fc domain by extensive alanine scanning of the solvent-exposed amino acid residues in CH2 and CH3 domains of human IgG1 [16]. Based on the binding profiles to the different FcγR, the IgG variants with single mutations can be categorized into different classes. Combination of IgG variants was then tested for the additive effects. Herceptin IgG with S298A/E333A/K334A mutations showed much more potent Her2+ cell killing activity in *in vitro* assays.

Employing computational design algorithms and high throughput screening, Xencor identified a set of Fc variants showing improved affinity for human FcγRIIIA. The combination of S239D/I332E (DE) and S239D/A330L/I332E (DLE) contributed to enhanced ADCC function. [16,17]. The same group also identified G236A variant with selectively enhanced binding to FcγRIIa relative to FcγRIIb [18]. This novel variant shows a 15-fold improvement in FcγRIIa/FcγRIIb binding ratio and can mediate enhanced phagocytosis by macrophages. Interestingly, in this study, no effect on phagocytosis was observed when FcγRIIb was selectively blocked, even though FcγRIIb has been reported to inhibit macrophage phagocytosis by modulating the threshold of activation [19].

An alternative approach for improving Fc and FcγR interaction has focused on the glycosyl modifications on the antibody Fc domains. It is known that the glycosylated portions of the CH2 domains are bound by the FcγRs and the glycan composition at those sites greatly determines the magnitude of the Fc effector functions [20]. For instance, prevention of fucosylation of an antibody causes highly enhanced ADCC activity via its increased binding to the FcγRIIIa [21]. Stewart *et al*. [22] constructed a human Fcγ1 variant library by error-prone polymerase chain reaction, to select for variants with higher affinity to human FcγRIIIA. The identified lead clone has F243L mutation in CH2, which causes decreased fucose content in the oligosaccharide chains. This result is another example that modulation of oligosaccharide profile is an effective way to modulate the effector functions [20].

In opposite to cancer therapy where improving the binding of Fc to FcγRs and/or C1Q for activation of ADCC, ADCP and CDC is beneficial, some other diseases and inflammation-related situations prefer using antibodies which are unable to activate Fc effector functions. IgG4 which is unable to activate Fc effector functions has traditionally been used for this task, however, lately it has not been used as a prominent approach due to its unique ability to cause swapping of heavy chains between IgG4 *in vivo* (Fab-arm exchange) [23,24], although some studies have shown that several mutations in IgG4 hinge domain may prevent this *in vivo* arm exchange [25]. In order to reduce the activator efficacy of the Fc domain of IgG1, mutations of the key sites within Fc domain that mediate the interaction with Fcγ receptors and C1q is performed by Fc engineering methods. The mutation of these residues would eliminate or decrease the binding of the antibodies to Fcγ receptors and/or C1q. For example, the binding site of the mouse IgG2a Fc domain to C1q was first identified via alanine scanning by Duncan and Winter, and this involved a region covering the hinge and upper CH2 of the Fc domain [12,26]. Then, the mutations K322A, L234A and L235A on the IgG Fc domain were identified by the researchers at Scripps Research institute and Genmab, and they showed that their combination is sufficient to almost prevent the interactions with FcγR and C1q [27]. Similarly, three mutations, L234F, L235E, and P331S, (also called TM) later discovered by MedImmune also have a highly similar effect on the Fc interactions [28]. Other Fc mutations reported to reduce ADCC and ADCP include IgG4 F234A/L235A [29], IgG1 L234A/L235A/G237A [30] and others.

An alternative approach to decrease Fc effector function of a therapeutic antibody is to alter glycosylation at its asparagine 297 residue that is required for optimal FcR binding. The N297 point mutations cause loss of antibody binding to the FcRs [16,31], Additional methods of glycosyl alterations include the enzymatic degylcosylation of Fc domains [32], expressing recombinant antibodies in the presence of glycosylation inhibitors [33], and the bacterial expression of the antibody Fc domains [34,35].

## ANTIBODY PHARMACOKINETIC IMPROVEMENT

### Antibody pharmacokinetic improvement

Improving the pharmacokinetics of a therapeutic antibody allows its dosage to be lowered, enables a subcutaneous formulation to be developed and the cost to be reduced. The improvement also prolongs the dosing interval, which is more convenient for the patients. Clearance of a therapeutic antibody from body is usually caused by two major mechanisms: (i) nonspecific clearance when it is nonspecifically internalized into the cellular endosomes; (ii) antigen binding-mediated internalization and clearance.

### Reduction of nonspecific clearance

Igawa *et al*. [36] demonstrated that lowering the pI of an antibody successfully improved its half-life, suggesting that it is a plausible way to reduce nonspecific clearance of antibodies. Therefore, selection of low pI antibodies from a library of antibody variants (usually generated with random site mutagenesis) or from different humanization variants would be a potential method to select therapeutic antibodies with reduced nonspecific clearance rate. Previous studies have also shown that nonspecific clearance of IgG could be improved by Fc engineering aiming to increase Fc/FcRn interaction. By improving the binding between Fc and FcRn, Scientists from PDL BioPharma revealed that the T250Q/M428L double mutant results in approximate 2-fold increase in IgG half-life in rhesus monkeys [37], and Scientists from MedImmune have revealed mutations M252Y/S254T/T256E (YTE) contribute to an approximately 4-fold increase in IgG half-life in cynomolgus monkeys [38,39]. Furthermore, in the clinical trial of Motavizumab in healthy volunteers, YTE mutation showed a 71–86% decrease in clearance and a 2–4-fold of increase in half-life [40].

**Table 1:** Therapeutic antibody optimization strategies, methods and different technologies used for them

**Purpose of optimization**	**Optimization strategy**	**Optimization method**	**Technologies used**
Better safety	Humanization	Modifications in the antibody structure	Phage display
			Yeast display
			Grafting of CDR
			Grafting of SDR
	Deimmunization and tolerization	Modifications in the antibody structure	Identifying and removing T cell epitopes
			Identifying and removing B cell epitopes
			Identifying and removing MHC epitopes
Better efficacy	Affinity maturation	Modifications in the antibody Fv fragment	Random mutagenesis
			Targeted mutagenesis
			Chain shuffling
			*In silico* technologies
	Fc effector function improvement	Modification of Fc/FcR or Fc/C1Q interactions	Engineering amino acid mutations in the antibody’s Fc fragment
			Modification of the glycosylation status in the Fc fragment
Better developability	PK improvement	Reduction of non-specific clearance	Decrease of the antibody’s isoelectric point
		Reduction of antigen binding-mediated clearance	Increase of the antibody’s capacity to dissociate from the antigen in acidic environments Increase antibody’s binding at neutral pH, to FcRn and/or antigen
	Pharmaceutical property improvement	Improvement of thermostability	Modification of amino acids in V_H_/V_L_ interface
			Elimination of hydrophobic residues on the antibody surface
			Optimizing conserved amino acid residues
		Improvement of solubility	Decrease of the antibody’s surface hydrophobicity
			Altering the antibody’s isoelectric point
			Engineering the antibody with N-linked glycan introduction within a CDR sequence
		Improvement of chemical stability	Prevention of antibody deamidation
			Avoiding antibody isomerization
			Prevention of succinimide formation
			Prevention of methionine and tryptophan oxidation
			Avoiding cysteinylation of unpaired cysteines in the CDR region
		Reduction of heterogeneity	Prevention of antibody glycosylation and *N*-pyroglutamine cyclization

### Reduction of antigen binding-mediated clearance

To reduce antigen binding-mediated antibody internalization and clearance, pH sensitive antibodies that interact with antigens at physiological conditions, but dissociate from antigens at low pH compartments (such as endosomes that have a pH of 6.0) could be generated or selected. There are two methods to develop pH-sensitive antibodies. The first method is to select pH-sensitive antibodies from a library of antibody variants or humanization variants, similar to the methods used to identify antibodies with reduced nonspecific clearance. The second method is called ‘His scan’, which is similar to the ‘Ala scan’ method, relying on the rational introduction of ionizable groups in the protein–protein interface. Murtaugh *et al*. [41] developed a combinatorial histidine library of antibodies with multiple ionizable groups, which changed the pKa on protein binding and then successfully selected antibodies that are highly sensitive to the pH changes but still retaining near wild-type affinity.

Igawa *et al*. [42] developed the sweeping technology, which employs engineering of the constant region of the antibody to increase its FcRn binding at neutral pH, subsequently enhancing the FcRn-mediated uptake of the antibody–antigen complex into the endosome where the antibody–antigen interaction is disrupted due to the low pH resulting in the antibody escape from degradation. This process would increase the number of cycles of antigen binding of the antibody before it undergoes the lysosomal degradation, thereby significantly improving both its pharmacokinetics (PK) and pharmacodynamics (PD). Fukuzawa *et al*. [43] developed recycling antibodies with characteristic variable region engineering, which have increased clearance of the antigen instead of the antibody, to improve their PK/PD profile.

## ANTIBODY PHARMACEUTICAL PROPERTIES IMPROVEMENT

Desirable pharmaceutical properties of a therapeutic antibody include: high thermostability, high solubility, high chemical stability and low heterogeneity. These properties contribute to the successful retention of biological activity of an antibody during storage, minimizing aggregation for less immunogenicity, improving production efficiency/yield to reduce cost of goods, enabling high concentration formulation and facilitating good quality control in manufacturing.

### Improvement of thermostability

Poor thermostability may cause antibody aggregation and low expression. In order to study the properties that affect protein thermostability, Vogt and Argos [44] examined 16 protein families with different thermostability properties, including the number and type of hydrogen bonds and salt links, polar surface composition, internal cavities and packing densities and secondary structural composition and revealed that the protein thermostability has a consistent positive correlation with the number of hydrogen bonds and polar surface area fraction. Therefore, to improve thermostability of an antibody, hydrophobic core and charge cluster residues need to be optimized. Library-based site mutagenesis study is one of the main approaches used for this optimization process. Several other approaches used to enhance the thermostability of the therapeutic antibodies are described in detail in the report by McConnell *et al*. [45]. These approaches include CDR grafting onto known antibody frameworks (using CDRs with defined specificity), consensus design in antibody variable domains that utilizes naturally occurring noncanonical regions of the antibody variants that improves the thermostability of the candidate antibody, computer-aided antibody structure modifications that optimize antibody thermostability *in silico* and protein stabilization by introducing mutations that form non-naturally occurring disulfide bonds between antibody domains (intradomain disulfide bonds in the case of camelid VHHs) [45].

### Improvement of solubility

High concentration of formulation is important for application of therapeutic antibodies in patients, especially for the treatment of chronic diseases. Because the volume for a single subcutaneous administration is generally limited to <1.5 mL, subcutaneous injection of antibodies often requires a formulation with high antibody concentration. This requires the clinical candidate antibodies to have a high solubility and very low viscosity. Therefore, the improvement of antibody solubility is critical for therapeutic antibody development. The intrinsic properties of antibodies play important roles in protein solubility, including size, hydrophobicity, electrostatic and charge distribution, among others [46].

Removal of surface hydrophobicity is a major approach for improving antibody solubility. Similar approaches used to optimize thermal stability such as structure-based engineering, including altering the pI and reintroduction of N-linked carbohydrate moieties into CDRs, can also be used to improve solubility of therapeutic antibodies [46].

A combination of structure-based design and somatic variant optimization can lead to substantially improved solubility while still retaining similar potency to the parent antibody, as exemplified by the solubility optimization of the therapeutic antibody by Kwon *et al* [47].

### Improvement of chemical stability

A lot of chemical degradation reactions can affect the antibody stability and lead to a reduction in the potency of a therapeutic antibody. For example, deamidation of asparagine (Asn) residues to aspartate (Asp) or isoaspartate (isoAsp) residues can lead to degradation during the CMC and storage [48]. When the deamidation of one to several Asn residues arises in the CDRs, the antibody’s binding potency will often be reduced [49–52]. The oxidation of antibody methionine residues at the Fc region can reduce its binding potency to the neonatal Fc receptor [53–56], whereas the oxidation of antibody tryptophan residues at CDRs has been reported to reduce the antibody’s binding potency to antigen [57–59]. Cysteinylation of unpaired cysteines in the CDR often lead to a reduction in the binding potency, stability and homogeneity of a therapeutic antibody [60,61].

Therefore, using antibodies with such tendencies as clinical candidates should be minimized; or if selected as a clinical candidate because of biological efficacy, the degradation sites on the protein structure surface need to be removed. Therefore, a crystal structure or a computationally generated 3D model structure could be used to study surface chemical degradation sites. Targeted site mutagenesis via a phage/yeast/mammalian library or computer-aided design can be used to remove those sites. For example, BioLuminate can be used to screen deamidation, oxidation and proteolysis sites on a 3D structure, followed by the targeted site mutagenesis to remove those sites.

### Reduction of heterogeneity

Heterogeneities caused by the posttranslational modifications such as glycosylation and *N*-pyroglutamine cyclization can lead to major inconsistencies of antibody quality from batch to batch [62–69]. Antibodies with these modifications need extra quality control in manufacturing, which increases their production costs. If cannot be avoided, the modification sites on those antibodies need to be removed via either targeted site mutagenesis or replacement by a variant residue from the antibody’s homologs generated from its antigen immunized antibody repertoire (unpublished data).

**Figure 3:**
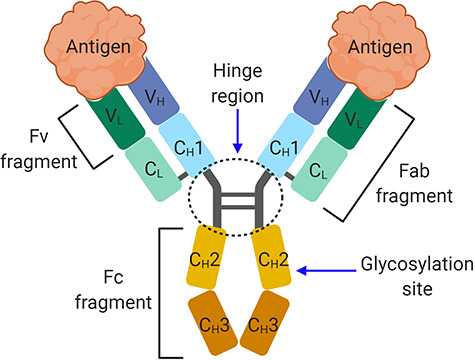
The general structure of a human IgG antibody. The domain organization in a human IgG antibody. The antibody structure is composed of two Fab fragments that mediate antigen binding and a Fc fragment that binds to immune cells, mediating Fc effector functions such as ADCC, ADCP and CDC.

The antibody optimization strategies, methods and technologies we have discussed in this review are summarized in Table 1. In addition, the general structural organization of a human IgG is shown in Figure 3 for reference.

*Conflict of interest statement*. All authors are current employees at Ab Studio Inc. Yue Liu is also the Founder of Ab Therapeutics Inc. Both Ab Studio Inc. and Ab Therapeutics Inc. participate in the commercial development of therapeutic antibodies.
